# Mitochondria-derived vesicles in neurodegeneration

**DOI:** 10.4103/NRR.NRR-D-25-00305

**Published:** 2025-06-19

**Authors:** Emanuele Marzetti, Riccardo Calvani, Hélio José Coelho-Júnior, Anna Picca

**Affiliations:** Fondazione Policlinico Universitario “Agostino Gemelli” IRCCS, Rome, Italy; Department of Geriatrics, Orthopedics and Rheumatology, Università Cattolica del Sacro Cuore, Rome, Italy; Department of Medicine and Surgery, LUM University, Casamassima, Italy

Mitophagy is a well-characterized and redundant recycling system for damaged mitochondria and a marker of organelle quality (Picca et al., 2023). Yet, the assessment of mitophagy *in vivo* remains a challenge. The characterization of the endosomal-lysosomal pathways supporting the endocytic trafficking has provided invaluable information also into mitophagy signaling. The endocytic pathway has been implicated in preserving mitochondrial quality via generation of mitochondria-derived vesicles (MDVs) and, as such, has been related to mitophagy tasks (Ferrucci et al., 2024). Altered mitophagy and MDV signaling accompany brain aging and neurodegenerative conditions (Ferrucci et al., 2024). However, how MDVs can be best characterized to be exploited as hallmarks of health and disease is debated. MDVs may be a *trait d’union* between dysfunctional mitophagy and decline of cell homeostasis through shuttling and/or being themselves mitochondria-derived damage-associated molecular patterns. These latter by instigating chronic low-grade inflammation may support neuroinflammation and neurodegeneration (Ferrucci et al., 2024). Alternatively, MDVs may rescue mitochondrial bioenergetics of neighbouring cells and favour neuronal health by transferring functional organelles. However, what defines one or the other role of MDVs and whether the outcome is mediated by vesicle subpopulations released under different metabolic triggers remain to be defined. Herein, we discuss MDVs as surrogate and more accessible measures of mitophagy. We also highlight the importance of addressing challenges in MDVs isolation and characterization to appreciate their signaling roles in neurodegeneration.

**Figure 1:**
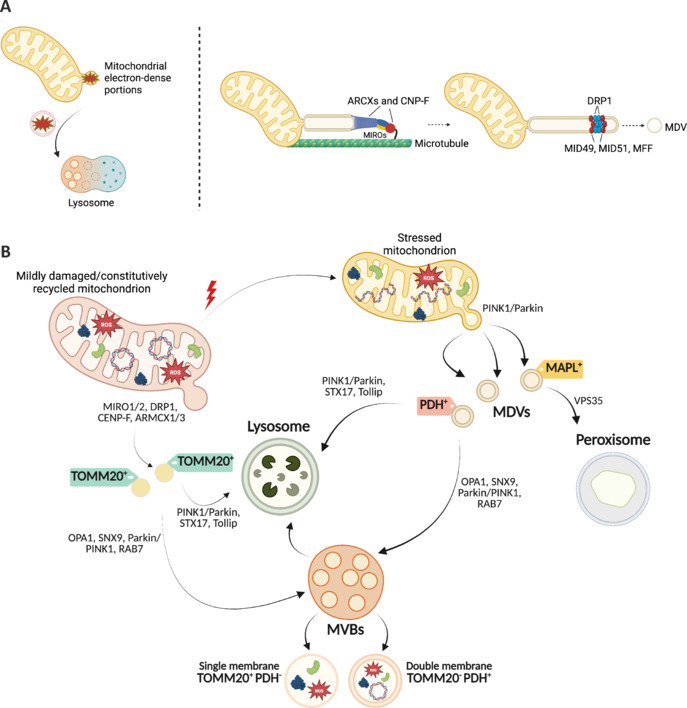
Cellular pathways involved in the formation of mitochondria-derived vesicles. (A) Pathways contributing to constitutive MDV generation. (B) Pathways for MDV generation according to the metabolic states of cells. Partially adapted from Ferrucci et al. (2024). Created with BioRender.com. ARMCX1/3: Armadillo repeat-containing proteins on the X chromosome 1/3; CENP-F: centromere protein F; DRP1: dynamin-related protein 1; MAPL: mitochondrial-anchored protein ligase; MDV: mitochondria-derived vesicle; MFF: mitochondrial fission factor; MID49 and MID51: mitochondrial dynamics proteins of 49 and 51 kDa; MIRO: mitochondrial Rho GTPase; MVBs: multivesicular bodies; OPA1: optic atrophy 1; PDH: pyruvate dehydrogenase; PINK1: PTEN induced kinase 1; RAB7: Ras-related protein in Brain 7; ROS: reactive oxygen species; SNX9: sorting nexin 9; STX17: syntaxin-17; TOMM: translocase of the outer mitochondrial membrane; VPS35: vacuolar protein sorting ortholog 35.

**Mechanisms of mitochondria-derived vesicle generation:** MDVs are a heterogeneous set of membrane-bound organellar structures originating from the outer and/or inner mitochondrial membranes and selectively encapsulating specific mitochondrial constituents (König and McBride, 2024). Two not-mutually exclusive mechanisms constitutively generate MDVs (**Figure 1A** and **B**). The first produces MDVs as mitochondrial electron-dense portions that are subsequently released via the vesicular pathway (König and McBride, 2024). The second generates MDVs as thin mitochondrial membrane protrusions along microtubules that are then portioned at their tips (König and McBride, 2024; **Figure 1A**).

In addition to the constitutive pathways, alternative routes have been identified according to the metabolic states of cells. Under stressful conditions, the PTEN-induced kinase 1 (PINK1)/Parkin(mitophagy)-dependent pathway is pursued (Ferrucci et al., 2024; König and McBride, 2024). The dynamin-related protein 1 (DRP1)(dynamics)dependent pathway, instead, is followed during steady states (Ferrucci et al., 2024; König and McBride, 2024).

In support of PINK1/Parkin-dependent MDV generation, evidence shows that cells treated with low doses of antimycin A, an inhibitor of mitochondrial complex III inducing mitophagy at high concentrations, release MDVs via PINK1/Parkin mediation as an early response upon stress induction (McLelland et al., 2014). Precursors of oligodendrocytes administered with low dose of carbon monoxide also produce PINK1/Parkin-mediated MDVs (Guo et al., 2023). Thus, PINK1/Parkin-mediated signaling may trigger MDV formation as an early quality control mechanism, selectively removing damaged mitochondrial components and/or facilitating cellular adaptation to mild mitochondrial stress. This process may precede and potentially delay the activation of mitophagy, which is typically engaged in response to more extensive mitochondrial damage. Indeed, activation of autophagy is not needed for MDV formation (McLelland et al., 2014). Conversely, the generation of vesicles carrying subunits of the matrix mitochondrial pyruvate dehydrogenase (PDH) and void of translocase of the outer mitochondrial membrane (TOMM)^−^/PDH^+^ has been observed under oxidative stress. Such vesicles differ from the most abundant TOMM^+^/PDH^−^ MDVs produced during the steady state and unload their contents to multivesicular bodies/lysosomes for elimination (McLelland et al., 2014; König and McBride, 2024; **Figure 1B**).

The PINK1/Parkin-independent pathway is responsible for the constitutive MDV biogenesis involving the microtubule-associated motor mitochondrial Rho GTPase 1 (MIRO1) and MIRO2 and DRP1, a mitochondrial dynamics mediator. While MIROs assist in the formation of mitochondrial tubules and cargo separation, DRP1 supports tubule scission and MDV generation (König and McBride, 2024). Conversely, the mitochondrial fusion mediator optic atrophy 1 enables MDV incorporation of mitochondrial matrix and inner membrane cargoes (Ferrucci et al., 2024; König and McBride, 2024).

Following their formation, MDVs can undergo distinct trafficking routes, being targeted to multivesicular bodies/lysosomes or peroxisomes for degradation, or alternatively, be secreted into the extracellular milieu (Ferrucci et al., 2024; König and McBride, 2024). The choice of these different routes is also complex, likely depending on specific stressors and type/degree of damage, and involves multiple mediators. PINK1, Parkin, Tollip, and syntaxin-17 are part of the signaling route directing MDVs to lysosomes, while the vacuolar protein sorting ortholog 35 (VPS35) and mitochondrial-anchored protein ligase are responsible for addressing MDVs to peroxisomes (McLelland et al., 2014; **Figure 1B**).

Conversely, inhibition of Parkin and guidance by cluster of differentiation 38 (CD38)/cyclic ADP ribose, sorting nexin 9, and optic atrophy 1 can drive MDV fusion with multivesicular bodies and their release at the extracellular level/bloodstream as extracellular vesicles (EVs). Finally, a subpopulation of EVs called “mitovesicles” has been identified with an unknown mechanism of release (D’Acunzo et al., 2022). Mitovesicles carry the voltage-dependent anion channel, cytochrome c oxidase subunit 4, and PDH-E1α (D’Acunzo et al., 2022), while they lack constitutive mitochondrial structures and proteins (D’Acunzo et al., 2022).

The vast array and heterogeneity of the pathways generating EVs are reflected by the wide range of EV types and composition. The incomplete knowledge of their generating mechanisms hampers a clear classification and the development of standard methods for their isolation (Ferrucci et al., 2024).

**Mitochondria-derived vesicle signaling in neurodegeneration:** Neuronal populations form dense synaptic connections whose basal activity is highly dependent on mitochondrial bioenergetics. Mitochondrial stress has been implicated in Parkinson’s disease (PD)–associated neurodegeneration (Ferrucci et al., 2024). Genetic mutations in the two mitophagy mediators PINK1 and Parkin have been described in PD. Lysosomal dysfunction has also been included among the molecular determinants of PD pathophysiology, mostly by favoring alpha-synuclein propagation (Ferrucci et al., 2024). Moreover, altered mitochondrial dynamics via DRP1 and fission protein 1–driven fragmentation have also been recognized as contributing factors to PD neurodegeneration (Ferrucci et al., 2024). Dynamin-like protein 1 (DLP1) localizes at the outer mitochondrial membrane and its turnover is mediated by the autophagy and retromer complex mediator VPS35 (Ferrucci et al., 2024). VPS35 is also implicated in MDV production by facilitating the trafficking of mitochondrial components either to lysosomes or peroxisomes (Ferrucci et al., 2024). When DLP1 interacts with the retromer complex through its association with VPS35, DLP1 is removed from mitochondria and MDVs are addressed to lysosomes for elimination.

Such trafficking allows efficient mitochondrial fission and supports mitochondrial quality. VPS35 mutations have been described in sporadic PD whereby an interaction of VPS35 with DLP1 is promoted and culminates in DLP1 recycling via MDV trafficking and retromer activity. As a result, hyper-fissioned and dysfunctional mitochondria are produced. Although the contribution of mitochondrial quality control (i.e., mitochondrial dynamics and mitophagy) to PD neurodegeneration has been well characterized, in-depth analyses of circulating MDVs and their signaling roles in PD are scarce. The EXosomes in PArkiNson Disease study revealed an increase in circulating levels of small EVs in older adults with PD compared with controls (Picca et al., 2020). These vesicles were enriched with mitochondrial components of the electron transport chain whose content was reduced compared with controls. These findings suggest that high levels of MDVs may be generated in the setting of altered mitophagy and mitochondrial dysfunction and that circulating MDVs might serve as a biomarker of PD.

Reduced levels of electron transport chain constituents have also been identified in circulating MDVs from individuals with Alzheimer’s disease (AD) compared with cognitively healthy peers (Yao et al., 2021). Furthermore, EVs containing mitochondrial proteins together with coding and non-coding mitochondrial RNA were found to be expelled by astrocytes, microglia, and neurons stressed with H_2_O_2_ and exposed to amyloid-β aggregates (Kim et al., 2020). Therefore, EV secretion may be a mechanism triggered by oxidative stress and mitochondrial dysfunction that installs a pro-oxidant environment and supports AD pathogenesis via formation of amyloid-β plaques, Tau-neurofibrillary tangles, and ultimately neurodegeneration. Finally, high levels of platelet-derived EVs containing mitochondrial components have been described in individuals with sporadic and familial AD (D’Acunzo et al., 2022).

MDVs have also been attributed a role as predictive and disease monitoring biomarkers in rare neurodegenerative disorders such as Huntington’s disease and fragile X syndrome (Ferrucci et al., 2024). The analysis of neuronal EVs isolated from the plasma of individuals with Huntington’s disease and derangements of the endocytic pathways revealed the inclusion of mtDNA and other mitochondrial constituents in circulating EVs (Ferrucci et al., 2024). Functional and quantitative alterations of EVs have also been described in the post-mortem cerebellar and frontal cortex of individuals with fragile X-associated tremor/ataxia syndrome and circulating neuronal EVs of asymptomatic individuals carrying premutations in the fragile X messenger ribonucleoprotein1 gene (Ferrucci et al., 2024).

**Conclusion and future directions:** All the conditions discussed (e.g., PD, AD, Huntington’s disease, and fragile X syndrome) show a certain degree of altered mitophagy and accumulation of damage-associated molecular patterns (DAMPs) arising from reduced cell homeostasis and mitochondrial stress. Although the mechanisms and pathways through which mitochondria-derived DAMPs, including MDVs, are generated and released at the extracellular level have not been completely elucidated, the extent of mitochondrial damage/dysfunction may be key to cellular decision (Todkar et al., 2021). Indeed, by carrying DAMPs, MDVs can support both disease progression through the promotion of inflammation or mitigate oxidative stress burden by extracting oxidized mitochondrial components. Therefore, deciphering the molecular pathways guiding each of the two routes is of utmost importance to harness MDVs for diagnostic and therapeutic purposes. Finally, the specific timing, magnitude, and persistence of MDV generation across disease stages remain to be systematically investigated.
